# Serum Apelin Level Predicts the Major Adverse Cardiac Events in Patients With ST Elevation Myocardial Infarction Receiving Percutaneous Coronary Intervention

**DOI:** 10.1097/MD.0000000000000449

**Published:** 2015-01-30

**Authors:** Hai-Tao Liu, Mai Chen, Jin Yu, Wei-Jie Li, Ling Tao, Yan Li, Wen-Yi Guo, Hai-Chang Wang

**Affiliations:** From the Department of Cardiology, Xijing Hospital, Fourth Military Medical University, Xi’an, China

## Abstract

The cardiovascular profile of the apelin makes it a promising therapeutic target for heart failure and ischemic heart disease. However, it remains unknown whether apelin affect the clinical outcome of patients with ST elevation myocardial infarction (STEMI) and received percutaneous coronary intervention (PCI).

We enrolled a total of 120 patients with acute STEMI who underwent primary PCI. Serum apelin was detected. After PCI procedure, all patients were followed for 12 months. The follow-up end-point was occurrence of major adverse cardiovascular event (MACE).

Lower serum apelin levels (<0.54 ng/mL) was significantly associated with higher serum low density lipoprotein-cholesterol level, higher peak creatine kinase MB fraction (CK-MB) and peak troponin-I (TNI) levels, the number of obstructed vessels, and need for inotropic support. The incidence of MACE was significantly higher in the low apelin group (23 patients out of 67) than in the high apelin group (10 patients out of 75, *P* < 0.001). Kaplan–Meier analysis revealed that the MACE-free rate was significantly lower in the patients with low apelin than those with high apelin (*P* < 0.001, log rank test). The multivariate Cox proportional hazard analysis adjusted with the clinical and angiographic characteristic reveals that the serum low apelin is a predictor for MACE incidence (hazard ratio = 2.36, 95% confidence interval: 1.83–3.87, *P* = 0.004).

The finding of this study suggests that the serum apelin may be used as a marker to predict the MACE after PCI in patients with STEMI.

## INTRODUCTION

ST elevation myocardial infarction (STEMI) is a one of the leading causes of cardiovascular mortality and morbidity worldwide. The clinical application of revascularization technique, for example, percutaneous coronary intervention (PCI), is effective to save ischemic myocardium, however, acute heart failure, cardiac shock, and mechanical complications often occur after successful procedure.^1,2^ Many clinical, echocardiographic, and biochemical factors can affect the prognosis of STEMI. Currently, there is no reliable prognostic predictor for patients with STEMI. Thus, it is urgently needed to identify new markers to identify patients at high risk for adverse clinical end-points.

Apelin (APLN) is the endogenous ligand for the previously orphaned G protein-coupled receptor, APJ. Apelin is secreted by white adipose tissue and its expression has been identified in many tissues.^3,4^ Increased apelin expression has been found in cardiovascular tissues, including cardiomyocytes, vascular smooth muscle cells, and endothelial cells.^5^ Not only as a powerful vasodilator, Apelin increases contractility in failing cardiac muscle.^6^ The apelin/APJ system is involved in the pathological conditions of heart failure.^7,8^ Apelin protects myocardial injury induced by isoproterenol in rat model.^9^ In preclinical models, apelin reduces peripheral resistance and improves cardiac performance in rats with failing hearts.^10^

The cardiovascular profile of the apelin makes it a promising therapeutic target for heart failure and ischemic heart disease. However, whether APLN can affect the post-PCI prognosis of patients with STEMI remains unknown. In this study, we found that the pre-PCI serum Apelin level is closely associated with the clinical outcome in STEMI patients receiving PCI, suggesting the serum apelin may be used as a prognostic marker for post-PCI prognosis in STEMI patients.

## METHODS

We enrolled a total of 120 patients with STEMI who underwent primary PCI at our institution between May 2009 and January 2013. The diagnosis of acute STEMI was made at admission according to the clinical symptoms, typical ECG changes, and elevated cardiac biomarkers levels including creatine kinase (CK), CK-MB fraction (CK-MB), and troponin-I.^2^ The diagnosis of acute STEMI was also confirmed by demonstrating the culprit lesion by coronary angiography. Patients with unstable angina and non-ST elevation myocardial infarction (NSTEMI) were not included in this study. We also excluded patients with significant organic valvular heart disease, known history of coronary artery disease (CAD) or previous MI, malignancy, collagen vascular disease, chronic kidney and hepatic failure, pulmonary embolism, and chronic inflammation disease. Clinical characteristics of all patients, including age, sex, smoking status, blood pressure, diabetes mellitus, lipid profile, and family history of premature CAD were collected. We also included 55 healthy controls for the comparison of apelin levels between STEMI patients and controls. The study was performed in accordance with the principles stated in the Declaration of Helsinki and approved by the Ethics Committee in our hospital. Informed consent was obtained from all patients prior to the study.

### PCI Treatment and Follow-up

All patients received 300-mg aspirin and a loading dose of 600-mg clopidogrel prior to the procedure. A sheath was inserted via femoral approach and an intravenous bolus of unfractionated heparin at a dose of 70 IU/kg was administered. Coronary stenting directly, or after balloon angioplasty, was performed where eligible. Glycoprotein IIb–IIIa inhibitor (tirofiban) was administered at the preference of the operator. After the success of PCI procedure, all patients were hospitalized and were followed after discharge from hospital for 12 months. The follow-up end-point was the occurrence of major adverse cardiovascular event (MACE), including rehospitalization due to unstable angina, heart failure, recurrence of myocardial infarction, revascularization with PCI or coronary artery bypass grafting, ischemic stroke, and cardiovascular death.

### APLN Elisa Detection

Blood samples were drawn immediately prior to primary PCI and serum was isolated by centrifugation within 1 h at 2500*g* for 10 min, and stored at −80°C. Serum concentrations of apelin (human apelin-12) were assayed using commercially available enzyme immunoassay kits (Phoenix Pharmaceuticals, Belmont, CA). Protocol was as follows: add 50 μL/well of standard, sample, or positive control, 25 μL of primary antibody and 25 μL of biotinylated peptide. Incubate at room temperature (20°C–23°C) for 2 hours. Wash immunoplate 4 times with 350 μL/well of 1 × assay buffer. Add 100 μL/well of Streptavidin-HRP solution and incubate at room temperature for 1 h. Wash immunoplate 4 times with 350 μL/well of 1 × assay buffer. Add 100 μL/well of 3,3’, 5,5’-tetramethylbenzidine substrate solution and incubate at room temperature for 1 h. Terminate reaction with 100 μL/well of 2N HCl. The intra-assay coefficients of variation (CVs) were 5% for apelin, and <14% for ghrelin, whereas the inter-assay CVs were 14% and 7.5%, respectively. Measurement of high-sensitivity C-reactive protein (hs-CRP) was performed using a particle-enhanced immunoturbidimetric assay (Hitachi 917 analyzer; Boehringer Mannheim, Germany). The detection limit was 0.1 mg/L, with intra- and inter-assay CVs of 1.34% and 5.7%, respectively.

### Statistical Analyses

Continuous variables were given as mean ± SD; categorical variables were defined as percentage. Data were tested for normal distribution using the Kolmogorov–Smirnov test. The Student *t* test was used for the univariate analysis of normally distributed continuous numerical variables and Mann–Whitney *U*-test was used for non-normally distributed numerical variables, and the *χ*^2^ test for the categorical variables. Spearman's rank correlation coefficient was used to analyze the relationship between numerical and ordinal variables. For presentation of data, 2 groups were formed as above and below the median APLN level. Moreover, additional groups were made up according to the presence or absence of MACE. Logistic regression analysis was used for multivariate analysis of independent variables. Kaplan–Meier curves of MACE-free survival among the 2 groups were compared using the log-rank test. All tests of significance were 2-tailed. Statistical significance was defined as *P* < 0.05. The Statistical Program for Social Sciences (SPSS for windows 16, Inc., Chicago, IL) was used for all statistical calculations.

## RESULTS

The median values (25th–75th percentiles) of apelin concentrations in healthy control and STEMI patients were 1.03 ng/mL (0.34–2.15 ng/mL) and 0.54 ng/mL (0.11–1.76 ng/mL, *P* < 0.001). The baseline clinical characteristics of STEMI patients are presented in Table 1. Based on the median value of apelin concentration, patients were divided into 2 groups: the high apelin group (apelin level ≥0.54 ng/mL; n = 75 patients) and the low apelin group (apelin level <0.54 ng/mL, n = 67 patients). The baseline clinical characteristics were subgrouped based on the serum apelin levels (Table 1). Our data show that lower apelin levels were significantly associated with older age (*P* < 0.001), higher serum low density lipoprotein-cholesterol (LDL) level (*P* < 0.001), higher hs-CRP (*P* = 0.002), and higher peak CK-MB and TNI level (*P* = 0.037 and *P* = 0.002, respectively).

**TABLE 1:**
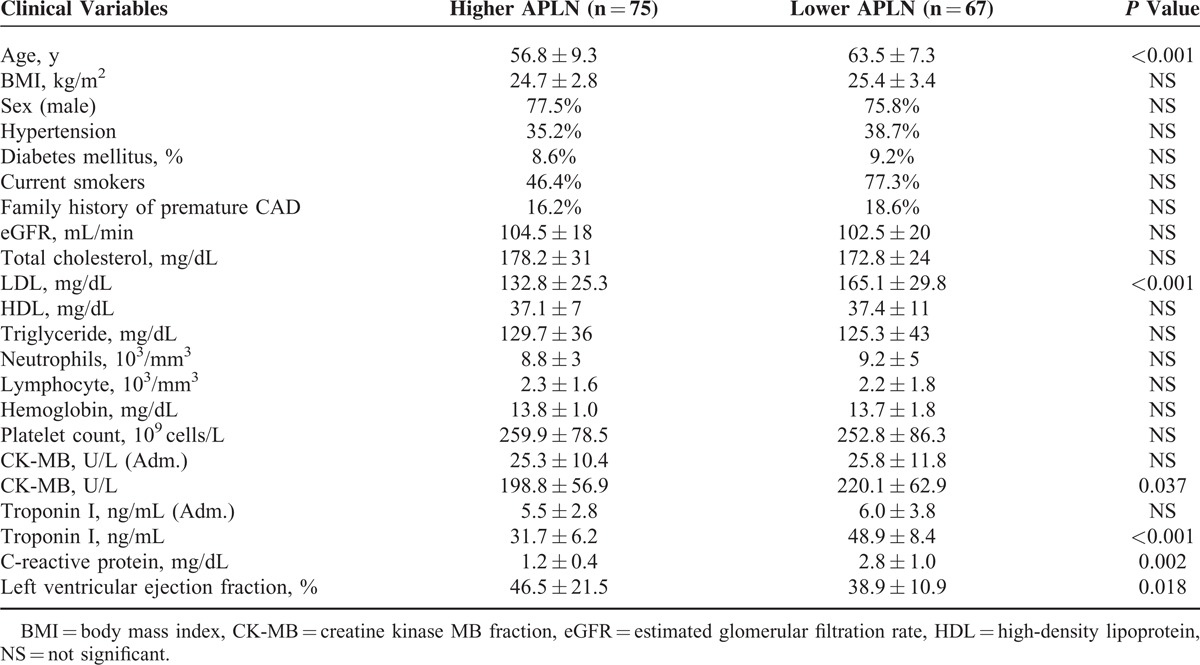
The Baseline Clinical Characteristics in Patients With Low and High APLN Levels

Angiographic characteristics and procedural results are detailed in Table 2. Lower apelin level was associated with the number of obstructed vessels (*P* < 0.001) and need for inotropic support (*P* = 0.013), but it is not related to the pain to balloon time, culprit vessel TIMI grade, culprit lesion location, culprit lesion length, stent number and types, and postprocedural TIMI flow grade (all *P* > 0.05, Table 2).

**TABLE 2:**
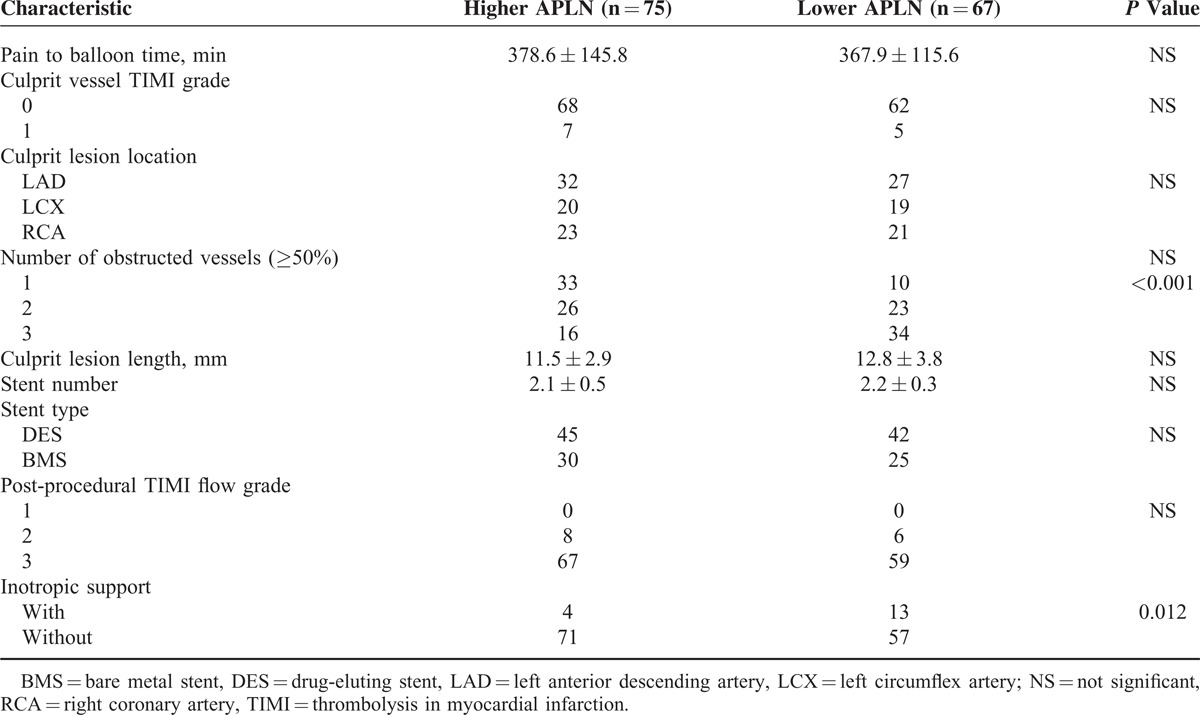
Angiographic Characteristics and Procedural Results of Patients With Low and High APLN Levels

**FIGURE 1:**
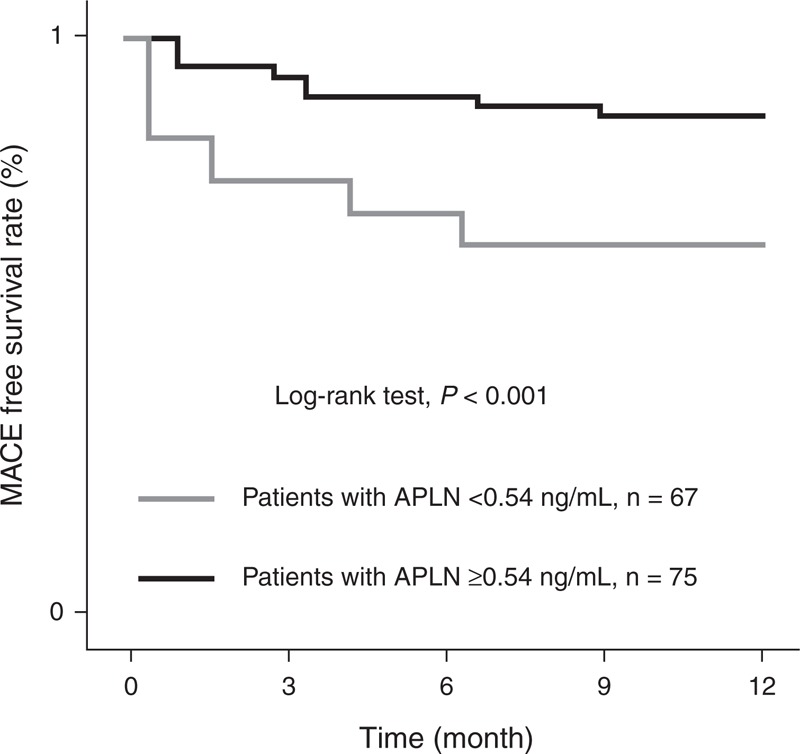
Kaplan–Meier analysis for MACE rate based on the serum apelin level of patients. This figure shows the MACE-free survival rates in the patients with low and high serum apelin levels. Our data show that low apelin serum level is significantly associated with lower MACE-free survival rate (*P* < 0.001, log rank analyses). MACE = major adverse cardiovascular event.

During the follow-up period, a total of 34 patents had MACE, including 6 cardiovascular deaths, 15 had recurrence of myocardial infarction, 11 had revascularization with PCI, and 2 had ischemic stroke (Table 3). The incidence of MACE was significantly higher in the low apelin group (23 patients out of 67) than in the high apelin group (10 patients out of 75, *P* < 0.001, Table 3). Kaplan–Meier analysis revealed that the MACE-free rate was significantly lower in the patients with low apelin than those with high apelin (*P* < 0.001, log rank analyses, Figure 1).

**TABLE 3:**
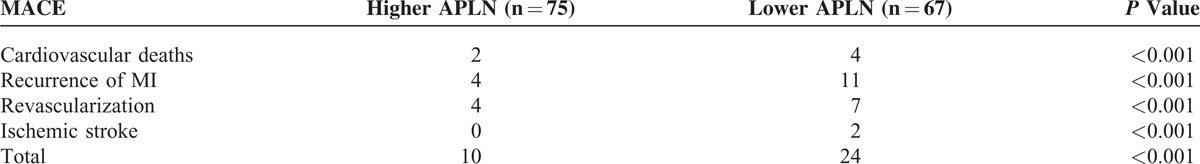
Incidence of MACE in Patients With Low and High APLN Levels 1 Year After PCI

To determine the predictor factors to the MACE incidence of these patients, we performed the multivariate Cox proportional hazard analysis and the variables that showed significance by univariate analysis were adopted as covariates. The data from baseline clinical and angiographic characteristics were included as cofounders. Our data reveal that low serum apelin is a predictor for MACE incidence (hazard ratio [HR] = 2.36, 95% confidence interval [CI] 1.83–3.87, *P* = 0.004). The other unfavorable prognostic predictor includes hs-CRP (HR = 1.81, 95% CI 1.11–3.02, *P* = 0.026), the need for inotropic support (HR = 2.02, 95% CI 1.75–3.45, *P* = 0.014), peak Troponin I (HR = 2.45, 95% CI 2.13–5.07, *P* = 0.002, Table 4).

**TABLE 4:**
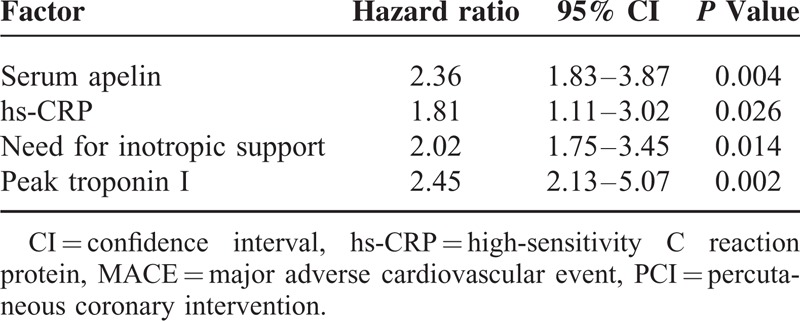
The prognostic factors for MACE in STEMI patients after PCI

## DISCUSSION

In this study, we explored the association between the serum apelin levels in patients with STEMI receiving PCI as the primary treatment. We found that the preprocedure serum apelin level is closely associated with important clinical features of these patients, including age, LDL, peak CK-MB and Troponin I levels, serum hs-CRP level, and number of obstructed vessels. More importantly, our follow-up study revealed that the serum apelin is significantly related to the incidence of MACE 12 months after successful PCI, suggesting that the serum apelin may be used as a molecular marker to predict the MACE after PCI in patients with STEMI.

The apelin-APJ system is emerging as an important regulator of cardiovascular homeostasis.^11–13^ To date, however, there is only limited evidence on apelin involvement in STEMI. Two groups from Poland found a significant decrease of plasma apelin-36 concentration in STEMI patients who reached composite end-point; nevertheless, they showed apelin-36 was insufficient to predict composite end-point in these patients.^14,15^

The result presented here provides evidence of a close relationship between apelin levels measured prior to primary PCI and in-hospital outcomes as well as long-term mortality. STEMI patients treated by primary PCI with low apelin levels depicted an unfavorable clinical profile (older age, higher LDL, hs-CRP, peak CK-MB and TNI values, and more obstructed vessels), and those patients also had higher incidences of MACE within the following 12-month, which is in accordance with the current evidence on the cardioprotective role of apelin.^16^ It is well known that apelin decreases in patients with severe left ventricular impairment^10^ and chronic heart failure,^7^ factors that highly influence the survival of STEMI patients. These findings alone could explain the worse clinical long-term outcome observed in patients with low levels of apelin. Moreover, after adjusting for other confounding clinical parameters, the association between apelin levels and morbidity rates still remained significant, indicating that apelin levels could provide independent predictive power for prognosis.

To our knowledge, this is the first study demonstrating that suppressed apelin levels are significantly associated with high incidence of MACE after primary PCI among patients with STEMI. In terms of a mechanism, apelin has been shown to cause vasodilatation in animal models as well as in clinical studies.^11,17,18^ Moreover, increasing evidence suggests a role for apelin in inflammation. For instance, apelin was reported to prevent aortic aneurysm formation by exerting a direct anti-inflammatory effect.^19,20^ Notably, our study showed serum apelin levels were inversely correlated with inflammatory marker, hs-CRP. Interestingly, a similar phenomenon has been observed in hemodialysis patients as well.^21^ Besides, apelin also plays roles in limiting the oxidative stress, which is a major component of myocardial reperfusion injury. Zeng et al showed that apelin may alleviate oxidative injury during reperfusion via decreasing generation of reactive oxygen species and increasing superoxide dismutase degradation.^22–24^ Furthermore, a previous report has demonstrated apelin's inotropic effects on normal as well as failing hearts.^25,26^ Indeed, in our STEMI population, patients with lower apelin levels needed more inotropic support after PCI compared with those with higher apelin levels.

Several limitations should be addressed in this study. First, this study included a relatively small cohort size, which may affect the statistical significance; therefore, a larger-scale study may be needed to more accurately assess the risk of future adverse events after PCI and to unravel the role of apelin in patients with STEMI. Second, analysis on all forms of apelin would be desirable in future investigations.

In conclusion, we report that patients with STEMI treated by primary PCI with lower levels of apelin are more likely to have worse prognosis compared with those with higher levels of apelin, suggesting a striking association between apelin levels and STEMI severity and MACE incidence. Our data indicate that apelin levels at admission may be considered as a useful marker for mortality risk stratification among STEMI patients. Understanding the role of apelin may have relevance to future preventive and/or therapeutic strategies for myocardial infarction.
